# Tumor heterogeneity and clinically invisible micrometastases in metastatic breast cancer—a call for enhanced surveillance strategies

**DOI:** 10.1038/s41698-024-00572-3

**Published:** 2024-03-29

**Authors:** Eliza R. Bacon, Kena Ihle, Weihua Guo, Colt A. Egelston, Diana L. Simons, Christina Wei, Lusine Tumyan, Daniel Schmolze, Peter P. Lee, James R. Waisman

**Affiliations:** 1 The Center for Precision Medicine, City of Hope National Medical Center, Duarte, CA USA; 2 Department of Medical Oncology, City of Hope National Medical Center, Duarte, CA USA; 3 Department of Immuno-Oncology, Beckman Research Institute at City of Hope, Duarte, CA USA; 4 Department of Pathology, City of Hope National Medical Center, Duarte, CA USA; 5 Diagnostic Radiology, City of Hope National Medical Center, Duarte, CA 91010 USA

**Keywords:** Breast cancer, Metastasis, Tumour heterogeneity, Breast cancer

## Abstract

The biology of metastatic breast cancer (MBC) is understudied, primarily due to the difficulty of procuring multiple samples from patients with oligometastatic breast cancer. We developed a rapid postmortem tissue procurement program that allows the collection and analysis of numerous metastatic lesions, subclinical locations, and potential pre-metastatic niches that fall within this scope. We conducted a rapid postmortem tissue collection study on 9 patients with MBC. Patients and their families consented to donate tissues immediately after death in an IRB-approved study. Various disease subtypes, progression histories, organ involvement, and final causes of death are reported. In patients with hormone receptor-positive (HR+) disease, estrogen receptor (ER), progesterone receptor (PR), HER2, and Ki-67 expression were heterogeneous across metastatic lesions within individual patients. Disease phenotype at the end of life trended toward complete loss of HR expression. Nearly all (*n* = 7) patients exhibited extensive tumor involvement of additional organs that had not been previously diagnosed clinically and were not retrospectively visible on recent imaging. Of these seven individuals, three included organs uncommonly associated with MBC: kidney, spleen, pancreas, and ovary. Finally, we identified clinically undetectable micrometastases in several organs uncommonly involved in MBC. Our findings raise several clinically relevant questions regarding the mechanisms of metastatic progression. Insights from this study argue for better surveillance strategies for monitoring MBC. We highlight the need to capture more accurate biomarker information in the context of heterogeneous disease and urge the consideration of treatment strategies that combine multiple targeted therapies.

## Introduction

Patients who have breast cancer rarely die as a result of their primary malignancy but, rather, due to their metastatic disease^1^. Despite this, individuals with advanced disease are often treated based on their primary tumor histology and its molecular and immunologic profiles. For these patients, infrequent re-biopsy of metastatic/recurrent sites may contribute to limited treatment efficacy. Furthermore, the unique genetic, phenotypic, and immune characteristics of metastatic tumors remain understudied, largely due to the challenge of obtaining metastatic tissue for research. Rapid postmortem tissue procurement (also known as rapid autopsy, warm autopsy, rapid tissue donation, etc.) enables the collection of research tissue from patients immediately after death. This approach provides a unique biospecimen resource to address questions regarding disease heterogeneity, and mechanisms driving disease progression^2^, and can provide clinically relevant disease information that is inaccessible while a patient is alive^3^.

Lymphatic and blood circulation are two of the main pathways through which cancer cells metastasize. In breast cancer, although there is abundant evidence demonstrating dissemination through blood^4^, the underlying mechanism of metastatic spread through lymphatics remains elusive^5^. Similarly, the timing in which breast cancer cell dissemination occurs is unclear; however, studies suggest that it can occur early in disease progression^6^. In metastatic breast cancer (MBC), distant tumor formation tends to occur in certain organs. This non-random phenomenon, known as “metastatic organotropism”^7^, is regulated by numerous factors: molecular features of cancer cells, host immune microenvironment, crosstalk, and interactions with local cells^8^. Still, it remains unknown if disseminated tumor cells (DTCs) can only seed these permissive tissues or if cells are disseminated globally but remain dormant in non-auspicious environments.

For women with MBC, the overall 5-year survival rate is 30%, but outcomes vary across molecular subtypes^1^ and by clinical presentation. Different subtypes of MBC differ in presentation, progression, and survival^9^. However, the effect of tumor heterogeneity on patient outcomes is less appreciated. The impact that these complexities have on therapeutic decision-making, response to therapy, and overall survival outcomes remains poorly understood. To assess systemic disease and explore tumor heterogeneity and dissemination in the context of MBC, we utilized a rapid postmortem tissue collection paradigm to systematically sample tumor and non-tumor specimens from nine patients at death. These samples were analyzed via immunohistochemistry and immunofluorescence to assess heterogeneity of clinical markers and to provide a comprehensive biological view of MBC.

## Results

### Clinical presentation of disease

**Fig. 1:**
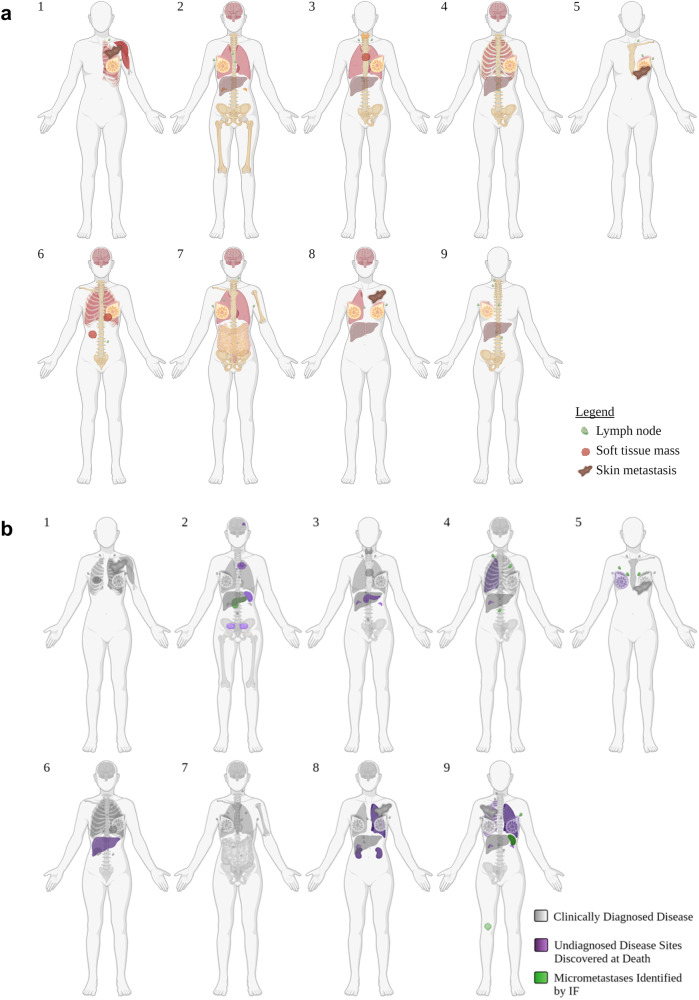
Diagnosed and unexpected disease. **a** Schematic of all clinically diagnosed sites of disease throughout each patient’s history up until death. **b** Clinically diagnosed disease sites shaded in gray. Purple shading indicates diseases identified at postmortem that were not previously diagnosed or suspected while the patient was alive. Green shading indicates the presence of micrometastases that were identified in “disease-free” tissues via immunofluorescence (IF) Figures created with BioRender.com.

Nine women with MBC were enrolled in this study. The clinical characteristics of their primary disease varied (Table 1), as did the clinical presentation of metastatic disease (Table 1; Fig. 1a). Cause of death was largely dependent on each patient’s unique circumstances of disease and the organs involved (Table 2). For example, patient 4 is believed to have died from causes unrelated to disease; patient 5 died due to terminal sedation; patient 6 participated in California’s End of Life Options Act; and patient 7 died from a therapeutic complication. Of note, patient 8’s medical history included two consecutive primary breast cancers: one ER-positive and one triple-negative breast cancer (TNBC).

**Table 1:** Patient characteristics and disease history

Disease characteristics at diagnosis							Metastatic disease history and unexpected post-mortem findings					
Patient	Age (years)	Race	Type	Subtype	Stage	TNM	Primary to metastatic Dx (months)	Metastatic Dx to death (months)	Primary Dx to death (months)	Clinically Diagnosed Metastatic Organ Involvement	Undiagnosed Findings Post-mortem	Micrometastases Identified by IF
1	46	Asian	IDC	Low HR / TNBC	IIA	T1N1a	19	10	29	L breast, Skin, Shoulder musculature, Chest wall, L Lung	N/A	N/A
2	31	Caucasian	IDC	HR+	IIA	T2N0	16	31	47	R breast, Lungs, Liver, Adrenals, Bone, Brain	Anterior Chest Mass, Spleen, Ovaries, Dura	Pancreas
3	32	Caucasian	IDC	HR+	IIIB	T3N1a	54	86	140	L breast, Anterior chest mass, Thyroid, Lungs, Liver, Bone	Pancreas, Adrenals	N/A
4	47	Middle Eastern	IDC	HR+	IIB	T2 N1a	125	163	284	L breast, Chest wall, Liver, Bone, Brain	R Lung, Adrenal	Non-tumor-draining lymph nodes
5	37	Caucasian	IDC	TNBC	III/IV	T4dN1	1	12	13	L breast, Skin, Bone	R Breast	Non-tumor-draining lymph nodes
6	67	Caucasian	IDC	Low HR /TNBC	II	T2N0	30	30	60	L Breast, Anterior Chest Mass, Chest wall, Lungs, Abdominal mass, Bone, Brain	Liver	N/A
7	58	Caucasian	Lobular	HR+	IV	N/A*	De novo metastatic	35	35	R breast, GI, Bone, Brain, Lung	N/A	N/A
8	69	Caucasian	IDC	HR+	I	T3mN0	27	7	34	L + R breast, Skin, R Lung, Liver, Brain	L Lung, R Adrenal, Kidneys	N/A
	69		IDC	TNBC	IIa	cT2cN0						
9	53	Asian	IDC	Low HR /TNBC	IIIC	cT2-3N3	7	6	13	R breast, Liver, Bone, Skin	L Breast, R Chest Wall, L Lung, Adrenals**	Spleen, Bone, Non-tumor-draining lymph nodes

*De novo metastatic with occult primary ** Not diagnosed but identified on imaging retrospectively; *IDC* invasive ductal carcinoma, *TNBC* triple-negative breast cancer, *HR* hormone receptor, *TNM* tumor, node, metastasis, *L* left, *R* right, *GI* gastro-intestinal.

**Table 2:** Tumor burden at time of death

Patient #	Lung	Liver	Skin	Brain	Bone	Cause of death
1	<1%	0%	25%	0%	<1%	Respiratory failure
2	<5%	95%	0%	<5%	25%	Liver failure
3	<1%	60%	0%	0%	35%	Liver failure
4	<1%	<1%	0%	0%	5%	Hemorrhagic stroke
5	0%	5%	20%	0%	0%	Terminal sedation
6	50%	<5%	0%	<5%	3%	EOLOA*
7	<5%	0%	0%	3%	<5%	Pulmonary hemorrhage during thoracentesis
8	5%	40%	10%	5%	0%	Multiorgan failure (brain, liver, & kidney)
9	<1%	65%	10%	0%	30%	Respiratory failure

Cause of death and percentage of organ with known metastases at the time of passing as assessed during tissue procurement and a review of the patients’ most recent clinical imaging results. *End of Life Options Act.

### Unexpected organ involvement at time of death

At the time of tissue procurement, seven of nine patients exhibited visible tumor in organs that were not previously identifiable on their most recent antemortem scan (Table 1; Fig. 1b). Four of these instances occurred in organs not commonly associated with breast cancer metastases, including pancreas, kidney, and ovary (Table 1 and Fig. 1b). In two other instances, bone specimens from sites presumed to have had a complete response to therapy (measured by the absence of FDG uptake on PET/CT imaging) were later determined to be >30% tumor-positive with a Ki-67 score of 10% suggesting moderately proliferating disease.

### Identification of micrometastases in disease-free organs

In-depth specimen collection numbers have been previously reported^2^. A total of 279 non-tumor specimens were collected from nine patients. A subset of these non-tumor tissues was then selected from each patient (an average of 10 specimens per patient) for further examination. All selected specimens were negative by prior clinical imaging, appeared grossly normal at procurement, and were reported to be tumor-negative by H&E assessment by a clinical breast cancer pathologist. Organs commonly and uncommonly involved in MBC were sampled, including the lung, bone, spleen, pancreas, kidney, and non-tumor-draining lymph nodes.

**Fig. 2:**
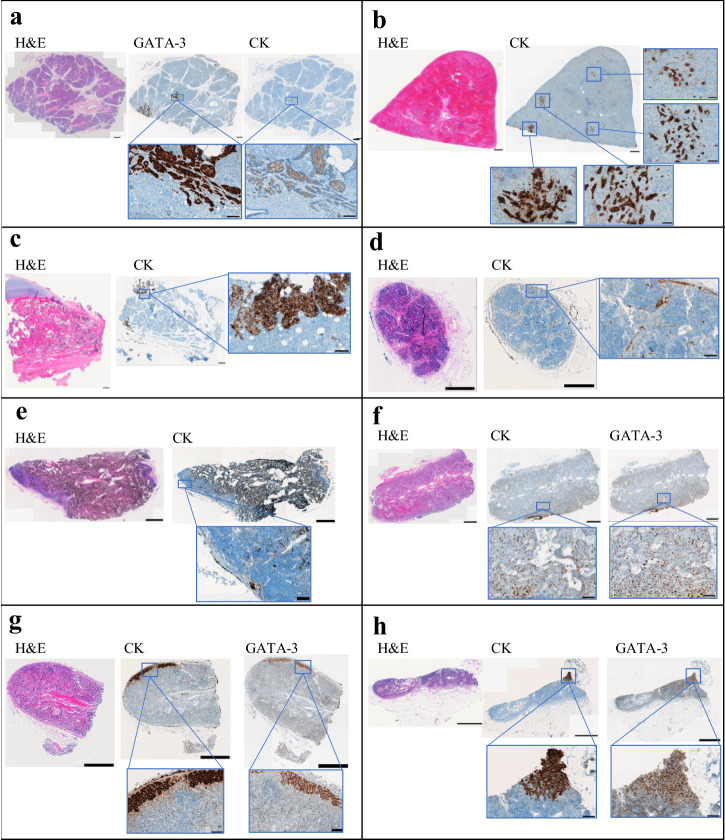
Identification of micrometastases in organs that rarely develop metastasis and in non-tumor-draining lymph nodes. Whole-slide scale bars have been standardized to 1000 μm. Magnified images scales have all been standardized to 100 μm. **a** Patient 2: pancreas. No clinical history of disease in pancreas or surrounding lymph nodes. At postmortem: grossly normal no adjacent positive lymph nodes. H&E: negative; GATA-3: positive; ER: positive. **b** Patient 9: spleen. No clinical history of disease in spleen; adjacent lymph nodes activity shortly before death. At postmortem: grossly normal, no adjacent positive tissues. H&E: negative; CK: positive. **c** Patient 9: Patella (bone). No clinical history of bone metastasis. At postmortem: grossly normal and no adjacent positive tissues. H&E: negative; CK: positive. **d** Patient 4: right supraclavicular lymph node. No history of disease; did not directly drain any cancer sites. At postmortem: grossly normal and no adjacent grossly positive tissues. H&E: negative; CK: positive; GATA-3: negative (data not shown). **e** Patient 4: left interlobular lymph node. No history of disease in lymph node or left lung. At postmortem: grossly normal and no adjacent positive tissues. H&E: negative; CK: positive; GATA-3: negative (data not shown). **f** Patient 5: right hilar lymph node. No history of disease to lymph nodes or adjacent tissues. At postmortem: grossly normal and no adjacent positive tissues. H&E: negative; CK: positive; GATA-3: positive. **g** Patient 5: right interlobular lymph node. No history of disease to lymph nodes or adjacent tissues. At postmortem: grossly normal and no adjacent positive tissues. H&E: negative; CK: positive; GATA-3: positive. **h** Patient 9: left axillary lymph node. No history of disease in lymph nodes. At postmortem: grossly normal and no adjacent positive tissues. H&E: negative; CK: positive; GATA-3: positive. (CK: cytokeratin).

Of the total 87 specimens assessed, we identified micrometastases in 13 specimens (15%) from four patients. Across these four individuals, micrometastases were found in the lung, bone, pancreas, spleen, and several non-tumor-draining lymph nodes (Fig. 2). More surprising was the identification of micrometastases in several lymph nodes that were not located anatomically downstream from a disease-involved organ. Image patterns demonstrated tumor cell infiltration into these lymph nodes within the subcapsular sinus (Fig. 2d–h). The presence of micrometastases in tumor-negative tissue did not correlate with tumor hormone status or histologic type. Combined with our findings at the time of procurement, unexpected and clinically undiagnosed metastases were detected in six of nine (67%) patients.

### Hormone receptor heterogeneity overtime and across metastatic sites

**Fig. 3:**
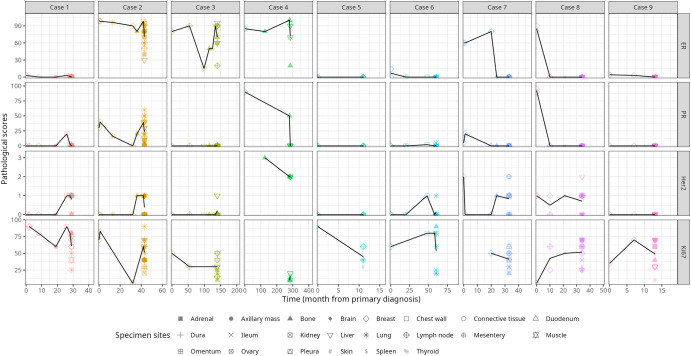
Hormone receptor (ER, PR, Her2) and tumor cell proliferation (Ki-67) changes during metastasis. Data variables from clinical biopsies were combined with post-mortem evaluations to create a visual depiction of changes in tumor marker expressions overtime, where 0 months represents a patient’s first biopsy. When multiple values are present at a single timepoint, the black trend line corresponds to the average of all values. Due to discrepancies in standard-of-care practices, not all historical data variables were available for all patients.

**Fig. 4:**
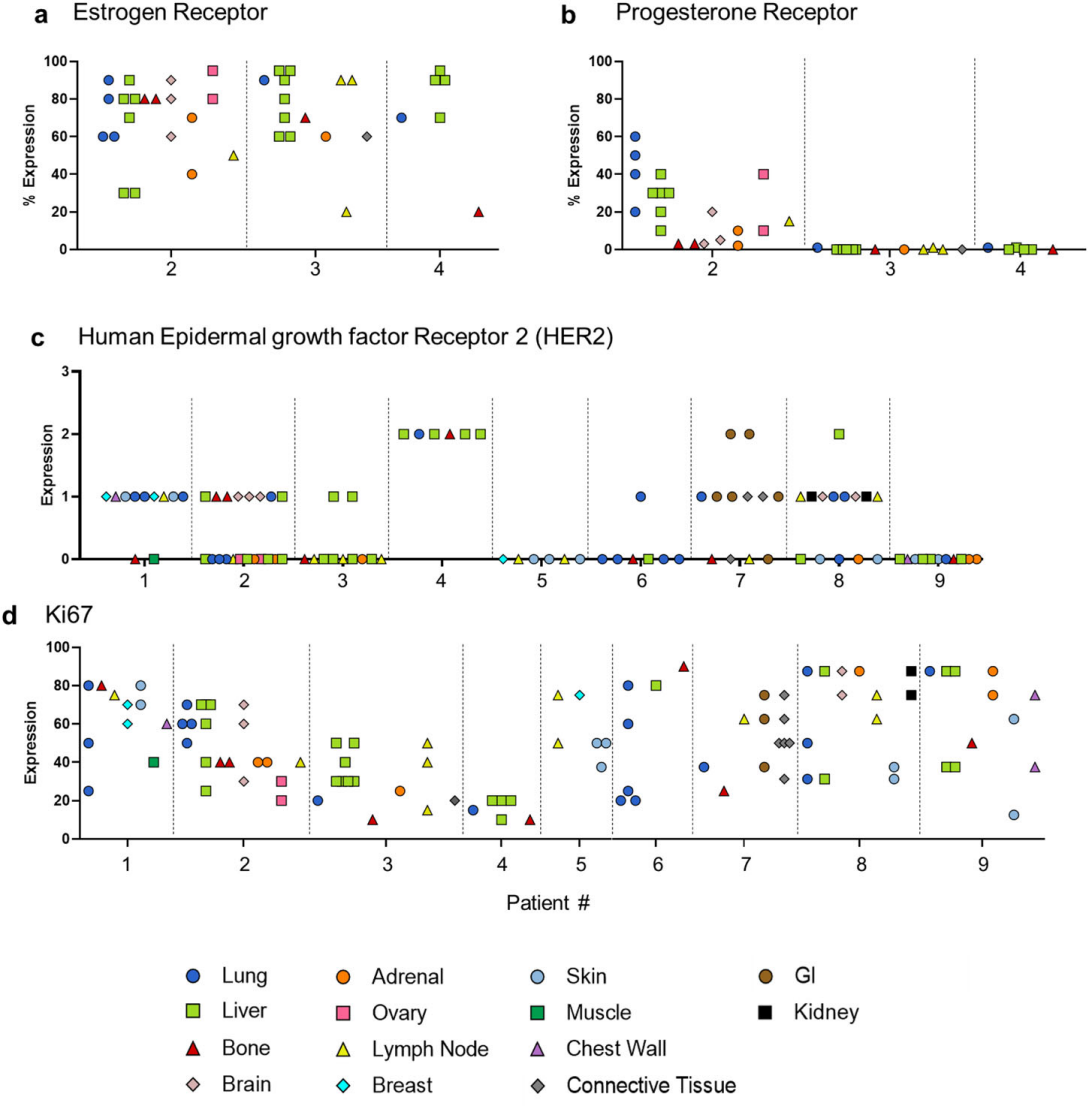
Hormone receptor and tumor cell proliferation (Ki-67) heterogeneity at postmortem. **a** Estrogen receptor (ER) expression in patients who maintained HR+ status at death. **b** Progesterone receptor expression in patients who maintained HR+ status at death. **c** Her2 expression across various metastatic sites across all patients. **d** Ki-67 expression across various metastatic sites across all patients.

A subset of tumor specimens was selected to assess heterogeneity in estrogen receptor (ER) and progesterone receptor (PR) hormone receptors. Data variables from clinical biopsies were combined with postmortem evaluation to create a visual depiction of changes in status over time and through disease progression (Fig. 3). Individuals with ER-positive primary breast cancer demonstrated variable ER and PR expression across time points and metastatic sites. Both ER and PR expression trended downward with time, transforming to a TNBC-like phenotype. Two HR+ patients (7 and 8) demonstrated a complete and sustained loss of HR expression. Three patients exhibited low levels of ER expression (1–12%) at diagnosis that either quickly dissipated or remained at extremely low levels (1–3%) during clinical progression before dissipating entirely at the end of life (patients 1, 6, and 9). In patients who remained ER+ at death (patients 2, 3, and 4), ER expression was profoundly heterogeneous both within and between organs (Fig. 4). PR expression did not correlate with ER status and was largely absent at the end-of-life, even when ER was still positive (Fig. 4b). Only one patient (patient 2) remained PR+ at death and demonstrated heterogeneous PR expression was both within and between organs.

### HER2 fluctuation overtime and gain of expression in late-stage disease

Of nine patients, six presented with 0+ HER2 score at the time of diagnosis; one patient had a score of 1+ (HER2-low), one patient had a score of 2+, and one patient demonstrated a score of 3+. Shockingly, of the six 0+ patients, four demonstrated a gain in expression on subsequent metastatic biopsies (Fig. 4c; patients 1, 2, 3, and 6). Most patients displayed modest heterogeneity, with scores fluctuating overtime between 0 to 1+. At the time of death, HER2 scores were largely heterogeneous across metastatic sites. (Fig. 4c).

### Ki-67 expression

Ki-67 scores were plotted per patient for each tumor overtime (Fig. 3). Expression varied throughout the course of the disease, with no clear trends. Expression of Ki-67 in late-stage disease was profoundly heterogeneous within each patient, both within and between organs (Fig. 4).

### PD-L1 expression across metastatic locations

**Fig. 5:**
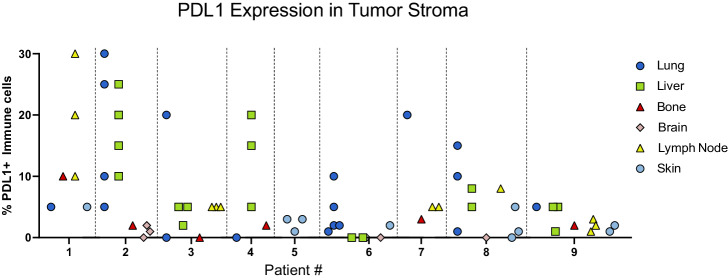
PD-L1 expression. The proportion of PD-L1 positive immune cells in the tumor stroma within tumor tissues collect at postmortem.

The proportion of PD-L1 positive immune cells within the tumor stroma demonstrated striking heterogeneity, both within and between metastatic sites, regardless of HR status (Fig. 5). A limitation of the study is that the Ventana PD-L1 SP263 is not considered a clinically validated antibody. However, for research purposes, we assume here that percent positive immune cells (% IC) is comparable to the clinical CPS score, which is defined as the percent of PD-L1–staining cells (tumor cells, lymphocytes, macrophages) relative to all viable tumor cells^10^. PD-L1 was highly (≥10%) and variably expressed in immune cells of both TNBC (patients 1, 6, and 8) and HR+ (patients 2, 3, 4, and 7) patients. All HR+ patients displayed at least one site with high PD-L1 expression. PD-L1 expression was homogenously low in only two patients (patients 5 and 9), both of which were TNBC. No significant relationships were observed to suggest an association between PD-L1 expression and organ type.

## Discussion

**Fig. 6:**
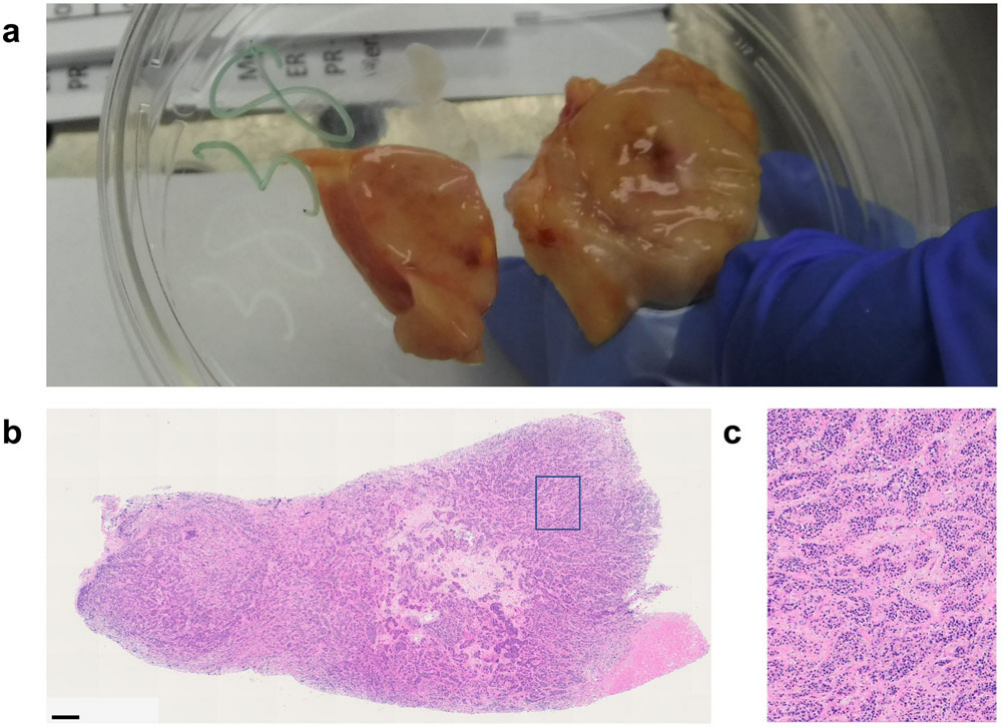
Tumor involved pancreas from patient 3. **a** Although not visible on clinical imaging, this large pancreatic tumor was grossly identifiable at post-mortem. This tumor (5.7 × 2.5 × 1.3 cm^3^) was unlikely to have developed during the one-month span of time between the last imaging study and death (Table 3). **b** H&E confirmed the specimen to be 100% tumor (as scored by a clinical, board-certified breast cancer pathologist. Scale bar: 600 μm. **c** ×20 magnification of H&E.

To better understand MBC, we expeditiously collected both metastatic and non-tumor tissues from patients who have died of active disease. In the postmortem setting, we expect to see increased tumor burden compared to that seen on clinical imaging and to find undiagnosed metastasis in organs that are commonly affected by MBC, such as the lungs or liver. In these instances, it is reasonable to assume that new involvement is explained by discontinued treatment and the rapid progression of disease that occurs at the end of life. However, the discovery of disease in uncommonly involved organs, such as the kidney, pancreas, spleen, and ovaries, is intriguing, especially considering the high proportion (3/9) of our patients with grossly visible tumors at these unexpected sites. Notably, each of these unexpected sites demonstrated extensive involvement that is unlikely to have developed during such a short window of time between the last imaging study and death (<1 month to 4 months) (Fig. 6; Table 3).

**Table 3:** Time since last imaging study from date of death

Patient #	Time since last scan	Type of imaging
1	<1 month	CT chest & abdomen
2	3 months	MRI abdomen/pelvis
3	1 month	CT abdomen/pelvis
4	2 months	CT chest, abdomen, pelvis
5	<2 months	Full body PET/CT
6	4 months	Full body PET/CT
7	<1 month	Full body PET/CT
8	3 months	Full body PET/CT
9	1 month	CT chest, abdomen, pelvis

Measured to the nearest months. Excludes local, organ-specific scans.

Breast cancer metastasis to the kidney, pancreas, or spleen is rarely diagnosed clinically and is most often discovered by chance at autopsy^11,12^. There is no consensus on the estimated prevalence of metastases to these organs in MBC. Pancreas involvement has been estimated to occur in 3–13% of cases^12–15^. A literature search revealed fewer than 50 cumulative case reports published between the years 1967 and 2022. Our findings lead us to question our assumptions about breast cancer organotropism and suggest that cancer cell colonization to these “rare” organs occurs more frequently than is recognized.

It is thought that the primary source of metastatic growth arises from a specialized group of cells, that spread from the main tumor site and settle in distant tissues, called DTCs^16^. DTCs can travel to distant organs and remain in a dormant state for an extended period of time before developing into visible metastases^17,18^. Unfortunately, detecting these small, disseminated cell populations is clinically difficult^19^. Herein this study, we identified clinically indiscernible micrometastases in rare organs in two-thirds of our patients. These sites were undetectable on routine pathologic H&E examination and subsequently identified by immunofluorescence. This provides insight into patterns of metastatic dissemination and suggests that breast cancer cells are capable of seeding indiscriminately throughout the body and only proliferate under auspicious conditions. Regarding the three patients with gross involvement in unexpected organs, we hypothesize that signaling from either the cancer cell itself or the microenvironment, may have allowed previously dormant DTCs to proliferate into visible tumors. Elucidating the specific mechanisms of tumor formation in these uncommon organs will provide insight into potential therapeutic strategies to prevent metastatic spread.

Our findings also support that breast cancer cell dissemination frequently occurs through lymphatics and can occur relatively early in the disease. The spread of cancer cells to regional, tumor-draining lymph nodes is an important prognostic factor in many cancer types^5^. In breast cancer specifically, lymphatic invasion is considered to be a predominant method of disease metastasis^20,21^. However, the migration of cancer cells into non-tumor-draining lymphatic vessels and the subsequent development of distant tumors, a process referred to as “skip metastasis,” has not been well studied in breast cancer. The prevalence of “skip metastasis” has been previously reported in other cancer types to occur in less than 25% of cases^22,23^; though, the mechanisms by which this occurs remain largely unknown^5^. Micrometastatic lesions in non-draining lymph nodes were identified in three of our nine patients (a total of seven nodes across three patients). All identified nodes shared similar features of metastatic invasion from the subcapsular zone inward, indicating that these cells did not enter through the blood vasculature. Moreover, our search was not exhaustive, and the number of specimens surveyed was limited. To what degree increased tumor burden plays a role in this phenomenon is unknown. However, two patients (patients 4 and 5), in whom we identified multiple non-draining nodes (Fig. 2d–g), died with a low tumor burden (Table 2) and from causes indirectly related to their cancer (Table 1). This suggests that cancer cell dissemination through lymphatics can occur relatively early in metastatic disease and may not merely reflect end-stage, high-tumor burden disease.

Undeniably, current clinical technologies cannot accurately identify micrometastases. This is particularly worrisome in the metastatic setting and reiterates the need for better tools to survey systemic disease. This is a relevant concept given the recent data from the NRG cooperative trials demonstrating that treating oligometastatic disease with local therapy (radiation or surgery for up to five sites) along with systemic therapy does not improve progression-free survival^24^. However, treatment strategies of this nature rely on the assumption that all areas of metastatic involvement are known. The failure of these trials, then, is unsurprising when considered in the context of clinically invisible, micrometastatic disease. It is unknown whether these “invisible” sites represent dormant cells with future metastatic potential or are a herald of a newly emerging, aggressive subclone. Regardless, if we can detect these sites early and treat them accordingly, we may indeed be able to prolong life.

Another major challenge in treating MBC is the heterogeneous nature of metastases^25,26^. Growing evidence indicates that tumor heterogeneity likely underlies mixed or incomplete response, as well as the eventual acquired resistance, to therapy that is commonly seen in MBC^27,28^. Both ER and PR positivity are independently correlated with favorable prognosis, and conversely, the decrease or loss of either receptor is associated with worsening clinical outcome^29–31^. Studies investigating clinical changes in HR expression have produced conflicting results; the degree and pattern of change have been inconsistent across studies^32^. Predominantly, however, these studies have all observed a decrease in expression, ranging from 24 to 38% of HR+ patients^32^. Interestingly, gain of HR expression is rarely observed and, when seen, is likely the result of underlying heterogeneity leading to discordant values between consecutive biopsies^32^. Our data here supports these findings and further suggests that, given enough time, all HR+ patients will experience a decrease or total loss of HR expression.

Loss of HR expression can occur spontaneously, through selective pressure of pre-existing subclones during treatment, or by therapy-induced epigenetic changes^33,34^. The timeline in which these changes occur is poorly understood. Our study suggests that loss of hormone status is likely to occur suddenly in ER-low populations and more gradually in those with a high % ER positivity. The impact that this timeline has on clinical management is not well appreciated, and the optimal frequency of HR reassessment is poorly defined in clinical guidelines.

Her2 overexpression/amplification is estimated to occur in 15–20% of all breast cancers and is associated with worse prognosis^35^. Loss of HER2+ is well documented^36–42^, both after chemotherapy (40%)^42^ and during the transition from primary to metastatic status (24%)^40^. How these changes influence prognosis is not clear. Conversely, gain of HER2 expression is estimated to occur in ~20% of the cases, mainly from HER2-0 to HER2-1 + /low, with these patients exhibiting lower rates of brain metastasis and increased disease-free survival^43^. Interestingly, gain in HER2 expression has been previously reported to occur more frequently in HR+ patients compared to those with TNBC^44^. In our limited cohort presented here, we observed fluctuations of Her2 expression in 8/10 patients; four of which experienced a gain in expression from HER2-0 to HER2-1 + /HER2-low. We did not detect any significant correlation between HER2 gain of expression and HR status.

Unfortunately, current standard-of-care procedures for evaluating progressing MBC are insufficient for capturing disease heterogeneity. The histological subtype of a tumor guides therapeutic decision-making. However, targeted therapies are often selected based on biomarker evaluation within a patient’s original tumor (or a very early metastatic biopsy), rather than from a real-time metastatic site. It is not surprising, then, that patients treated in this manner do not experience a robust response from tumor-directed targeted therapies. Furthermore, repeat, or subsequent biopsies of multiple metastatic sites are not universally practiced. As we present here, each distinct metastatic lesion can demonstrate a unique phenotype that evolves overtime. For example, loss of HR and gain in HER2 expression in some metastatic sites. This suggests that previously HER2-negative patients may still benefit from anti-HER2 therapy^45^ and additional biopsies should be taken periodically to reassess for gain of expression.

Similarly, nearly all patients exhibited PD-L1 expression that varied across metastatic sites. Had any of these patients received a biopsy in the clinic, their results (and the subsequent qualification/disqualification of targeted therapy) would have depended on the arbitrarily chosen biopsy site. Furthermore, although PD-L1 expression has been considered a feature more likely to be present in TNBC, we detected significant expression in all HR+ patients (patients 2, 3, and 4). This suggests to us that these individuals, as well as other advance HR + MBC patients, should be considered for treatment with immune checkpoint therapies.

Effectively treating heterogeneous disease will require new multifaceted therapeutic approaches. This may include conducting multiple biopsies at once and overtime in conjunction with combining systemic, targeted, and local therapy strategies. Additionally, enhanced therapeutic strategies that focus specifically on combining targeted therapies, could potentially maximize the number of metastatic sites being treated simultaneously. We recognize that many targeted therapies carry high toxicity risks, and we propose that, in the face of life-threatening disease, low-toxicity goals may need to be sacrificed for increased therapeutic effectiveness. While we argue that a single biopsy is insufficient to gain a comprehensive understanding of the systemic landscape of an individual’s heterogeneous metastatic disease, we acknowledge that biopsies are invasive procedures involving substantial risks and discomfort. Conducting multiple biopsies at once throughout the body is likely to be detrimental to the patient’s experience. Expecting patients to consent to a substantially increased biopsy schedule is unrealistic, and compliance is likely to be suboptimal. However, we argue that a modest increase in biopsy frequency (for example, at each progression event) is critical for enhanced therapeutic efficacy in MBC.

The meeting of tumor heterogeneity and clinically invisible disease is a dangerous one. We cannot effectively treat diseases that we can neither see nor evaluate. Our study clearly demonstrates that the current surveillance technologies are insufficient in identifying all metastatic sites. Overcoming this will require the utilization of alternative diagnostics, such as targeted imaging (e.g., ^18^F-fluoroestradiol PET, magnetic resonance spectroscopy (MRS) to detect LDHA activity), and the detection of circulating tumor DNA (ctDNA). While still in their infancy, these and other emerging techniques hold promise to enhance cancer care by potentially identifying the emergence or dissipation of specific tumor markers^46–49^ as well as improving the detection of disease in difficult-to-diagnose organs^50^. In the future, this could allow for dynamic adjustments to therapeutic strategies while avoiding the need for multiple biopsies^49,51^.

## Methods

### Study design

‘The Legacy Project’ for rapid tissue procurement and a detailed account of communication and the sequence of events were previously described^2^. The current study describes the collection and analysis of MBC patient data and biospecimens from the “Legacy Project”—a rapid tissue donation program at City of Hope. Nine women and their families were enrolled shortly before the end of life (1 week–6 months) or immediately after their death. Prior to death, written consent was obtained from the patient or from their next of kin at death. Health status was tracked in real-time through direct communication with the patient’s treating physicians and, when discharged, through the hospice team. At death, the attending nurse immediately notified the project coordinator, and transportation was arranged to retrieve the patient from the place of death. Within 1–2 hours after death, the patient was delivered to the autopsy facility, where tissues were procured within 6 hours of death. After the procedure was complete, the project coordinator arranged final transportation to the funeral home, and the family was notified. This study was conducted under City of Hope IRBs #17503 & #18352, in compliance with all relevant ethical regulations, including the Declaration of Helsinki.

### Clinical data collection

Patients’ medical charts were reviewed to obtain disease information, including dates of diagnoses, treatment histories, pathology reports, therapeutic histories, and clinical tumor markers. Data variables from clinical biopsies were combined with postmortem evaluation to create a visual depiction of changes overtime and through disease progression in each patient. Radiology imaging history was reviewed in depth by a clinical radiologist by assessing each diagnostically relevant scan, including staging PET/CT, CT, and MRI, and their corresponding reports, to determine known sites of disease as well as a detailed history of individual tumor growth and response to therapy over the patients’ disease course.

### Tissue collection

Tissue procurement was performed following standard autopsy procedures. Grossly tumor-positive organs and non-diseased tissues of high interest were thoroughly examined by dissection. For all tumor specimens, matched non-tumor or grossly normal tissue was taken, including adjacent and contralateral tissue when available and sites of “resolved” disease, depending on disease presentation. Organs with no clinically identifiable disease were also sampled, including non-grossly normal tissues. For disease-free organs, specimens were collected as close as possible to the organ’s major incoming artery.

Tumor tissues were identified using standard tumor indicators, including changes in tissue color, density, and texture. Organs or areas of complete response to therapy were identified using the clinical and imagining history as a guide. Tissues were collected as follows: specimens were bulk dissected from the organ or location of interest as a ~2 cm^3^ square containing tumor and normal tissue, placed in a sterile petri dish, and photographed.

### Tissue processing

Tumor tissues placed in cassettes for formalin-fixed paraffin-embedded (FFPE) processing were grossed, leaving a “tag” of normal tissue to assist in the pathologist’s validation review. All surrounding normal tissue for tumor samples placed in conical tubes or cryovials were removed. Tissue cassettes were placed immediately into 10% neutral buffered formalin for a minimum of 48 hours. Dehydration, clearing, and paraffinization were performed on a Tissue-Tek VIP Vacuum Infiltration Processor (SAKURA). Samples were embedded in paraffin using a Tissue-Tek TEC Tissue Embedding Station (SAKURA). For single-cell suspensions, tissues were placed into tubes containing cold HBSS (Life Technologies, Thermo Fisher Scientific) and transported on ice to the laboratory for processing within six hours of collection.

### Hematoxylin & eosin (H&E) staining

Tissue samples were sectioned at 5 µm and placed on positively charged glass slides. Slides were deparaffinized, rehydrated, and stained with Modified Mayer’s Hematoxylin and Eosin Y Stain (America MasterTech Scientific) on an H&E Auto Stainer (Prisma Plus Auto Stainer, SAKURA) according to standard laboratory procedures. Specimens were validated by a clinical board-certified breast cancer pathologist and scored for percent (%) tumor involvement, and % necrosis. Data from specimens containing >50% necrosis were excluded from analysis.

### Hormone receptor clinical panel

Tissue samples were sectioned at 4 μm, placed on positively charged glass slides, and baked. Slides were loaded on a Ventana Discovery Ultra (Ventana Medical Systems, Roche Diagnostics) automated immunohistochemistry (IHC) staining machine for deparaffinization, rehydration, endogenous peroxidase activity inhibition, and antigen retrieval (pH 8.5). Following each primary antibody incubation (ER, clone (SP1)250, Ventana, ready to use, cat#: 790-4325; PR, clone 1E2, Ventana, ready to use, cat#: 790-4296; HER2, clone 4B5, Ventana, ready to use, cat#: 790-2991; Ki-67, clone 30-9, Ventana, ready to use, cat#: 790-4286), DISCOVERY anti-Rabbit HQ and DISCOVERY anti-HQ-HRP were incubated. Stains were visualized with DISCOVERY ChromoMap DAB, counterstained with hematoxylin (Ventana), and coverslipped. Specimens were scored by a clinical board-certified breast cancer pathologist.

### PD-L1 and immune panel

Tissue samples were treated as described above. Antigens were sequentially detected, and heat inactivation was used to prevent antibody cross-reactivity between the same species. Following each primary antibody incubation (CD8, clone SP57, Ventana, ready to use, cat#: 790-4460; PD-L1, clone SP263, Ventana, ready to use, cat#: 740-4907; CD20, clone L26, Ventana, ready to use, cat#: 760-2531; CD68, clone PG-M1, DAKO, dilution 1:100, cat#: M0876; pan-CK, clone AE1/AE3/PK26, Ventana, ready to use; cat#: 760-2595), DISCOVERY anti-Rabbit HQ, DISCOVERY anti-Mouse HQ, and DISCOVERY anti-HQ-HRP were incubated. Stains were visualized with DISCOVERY Yellow Kit, DISCOVERY Teal Kit, and DISCOVERY Purple Kit, respectively, counterstained with hematoxylin (Ventana), and coverslipped. The proportion of PD-L1-positive immune cells within the tumor stroma was assessed by a clinical, board-certified breast cancer pathologist.

### Micro metastasis panels

Tissues were stained for one or more of the following markers: pan-CK, clone AE1/AE3/PK26, Ventana, ready to use; cat#: 760-2595; GATA-3, clone: L50-823, Ventana, ready to use, cat#: 760-4897; HMFG, clone: SPM291, abcam, dilution 1:400, cat#: ab17787; MUC1, clone: H23, Ventana, ready to use, cat#: 790-4574; and ER clone (SP1)250, Ventana, ready to use, cat#:790-4325 (if the patient was previously ER-positive), depending on tissue type. Immunohistochemistry was performed using the Ventana Discovery Ultra (Ventana Medical Systems, Roche Diagnostics, Indianapolis, USA) IHC automated Stainer. Briefly, slides were deparaffinized, rehydrated, and incubated with endogenous peroxidase activity inhibitors and antigen retrieval reagents. Each primary antibody was incubated, followed by DISCOVERY anti-rabbit HQ or DISCOVERY anti-mouse HQ, and DISCOVERY anti-HQ-HRP incubation. Stains were visualized with DISCOVERY ChromoMap DAB Kit, counterstained with hematoxylin (Ventana), and coverslipped.

### Visualization of pathological quantifications

To visualize the hormone receptor scores and Ki-67 percentage with collection time, ggplot2 (v3.3.6) on R (v4.1.3) was used to visualize all data points. The average of scores was calculated for the samples at the same collection time and shown by connected black lines.

### Reporting summary

Further information on research design is available in the Nature Research Reporting Summary linked to this article.

### Supplementary information

REPORTING SUMMARY

## Data Availability

The data generated in this study are available upon request from the corresponding author.
