# Supplementary material for: Tumor heterogeneity and clinically invisible micrometastases in metastatic breast cancer—a call for enhanced surveillance strategies
Source: NPJ Precis Oncol. 2024 Mar 29;8:81. doi: 10.1038/s41698-024-00572-3 (PMC10980766; doi:10.1038/s41698-024-00572-3)
Supplement: Supplementary file 1 — REPORTING SUMMARY [file 41698_2024_572_MOESM1_ESM.pdf]

nature portfolio

Corresponding author(s): Eliza R Bacon

Last updated by author(s): 2024/01/22

Reporting Summary

Nature Portfolio wishes to improve the reproducibility of the work that we publish. This form provides structure for consistency and transparency in reporting. For further information on Nature Portfolio policies, see our Editorial Policies and the Editorial Policy Checklist.

Statistics

For all statistical analyses, confirm that the following items are present in the figure legend, table legend, main text, or Methods section.

n/a	Confirmed	
☐	☒	The exact sample size (*n*) for each experimental group/condition, given as a discrete number and unit of measurement
☒	☐	A statement on whether measurements were taken from distinct samples or whether the same sample was measured repeatedly
☒	☐	The statistical test(s) used AND whether they are one- or two-sided<br>*Only common tests should be described solely by name; describe more complex techniques in the Methods section.*
☒	☐	A description of all covariates tested
☒	☐	A description of any assumptions or corrections, such as tests of normality and adjustment for multiple comparisons
☒	☐	A full description of the statistical parameters including central tendency (e.g. means) or other basic estimates (e.g. regression coefficient) AND variation (e.g. standard deviation) or associated estimates of uncertainty (e.g. confidence intervals)
☒	☐	For null hypothesis testing, the test statistic (e.g. *F*, *t*, *r*) with confidence intervals, effect sizes, degrees of freedom and *P* value noted<br>*Give P values as exact values whenever suitable.*
☒	☐	For Bayesian analysis, information on the choice of priors and Markov chain Monte Carlo settings
☒	☐	For hierarchical and complex designs, identification of the appropriate level for tests and full reporting of outcomes
☒	☐	Estimates of effect sizes (e.g. Cohen's *d*, Pearson's *r*), indicating how they were calculated

Our web collection on statistics for biologists contains articles on many of the points above.

Software and code

Policy information about availability of computer code

Data collection	No software was used for data collection.
Data analysis	To correlate the hormone receptor scores and Ki-67 percentage with collection time, ggplot (v3.3.6) was used to visualize all data points. The average of scores was calculated for the samples at the same collection time and shown by connected black lines. The visualization of correlation coefficients was implemented by the ggplot R package with customized scripts. R (v4.1.3) was used to calculate Spearman's correlation coefficient.

For manuscripts utilizing custom algorithms or software that are central to the research but not yet described in published literature, software must be made available to editors and reviewers. We strongly encourage code deposition in a community repository (e.g. GitHub). See the Nature Portfolio guidelines for submitting code & software for further information.

Data

Policy information about availability of data

All manuscripts must include a data availability statement. This statement should provide the following information, where applicable:

- Accession codes, unique identifiers, or web links for publicly available datasets
- A description of any restrictions on data availability
- For clinical datasets or third party data, please ensure that the statement adheres to our policy

The data generated in this study are available upon request from the corresponding author.

Research involving human participants, their data, or biological material

Policy information about studies with human participants or human data. See also policy information about sex, gender (identity/presentation), and sexual orientation and race, ethnicity and racism.

Reporting on sex and gender	This study only included female participants as the disease rarely occurs in males. We did not gather or report any data on gender.
Reporting on race, ethnicity, or other socially relevant groupings	Race was included in our summary of patient characteristics because breast cancer often presents with differing subtypes amongst various racial/ethnic groups. While we did not perform any analysis using this variable, we thought it was important to include as background information.
Population characteristics	This study includes 9 women with metastatic breast cancer. The clinical characteristics of their primary disease varied (Table 1), as did the clinical presentation of metastatic disease (Table 1; Figure 1A). Cause of death was largely dependent on each patient's unique circumstances of disease and the organs involved (Table 2).
Recruitment	Nine women and their families were enrolled shortly before the end of life (1 week–6 months) or immediately after their death. Prior to death, written consent was obtained from the patient or from their next of kin at death. Health status was tracked in real-time through direct communication with the patient's treating physicians and, when discharged, through the hospice team. At death, the attending nurse immediately notified the project coordinator, and transportation was arranged to retrieve the patient from the place of death.
Ethics oversight	This study was approved by City of Hope's Institutional Review Board under study numbers 17503 and 18352. Participants were enrolled either by self-consent prior to death, or had their body donated after death by next-of-kin.

Note that full information on the approval of the study protocol must also be provided in the manuscript.

Field-specific reporting

Please select the one below that is the best fit for your research. If you are not sure, read the appropriate sections before making your selection.

☒ Life sciences ☐ Behavioural & social sciences ☐ Ecological, evolutionary & environmental sciences

For a reference copy of the document with all sections, see nature.com/documents/nr-reporting-summary-flat.pdf

Life sciences study design

All studies must disclose on these points even when the disclosure is negative.

Sample size	The original study was conducted as a program "pilot" aimed to recruit 8-10 patients over an 18 month period. The data presented here represents 9 patients recruited during a 13 month time frame. Within each patient, multiple specimens were collected, with the exact number dependent on disease presentation. A total of 279 non-tumor specimens were collected from nine patients. In-depth specimen collection numbers have been previously reported [2].
Data exclusions	no data was excluded from the study.
Replication	Specimens collected from human subjects were each considered to be unique samples. However, multiple specimens were sampled within each organ and tumor within all patients. All data measurements were treated according to "standard-of-care "clinical processes and guidelines to ensure relevancy to the field. Specimens were read and data validated by two clinical, board certified pathologists.
Randomization	N/A
Blinding	Specimens were de-identified after collection and the clinical pathologists involved in scoring histological characteristics were blinded to any clinical characteristics associated with the specimens.

Reporting for specific materials, systems and methods

We require information from authors about some types of materials, experimental systems and methods used in many studies. Here, indicate whether each material, system or method listed is relevant to your study. If you are not sure if a list item applies to your research, read the appropriate section before selecting a response.

Materials & experimental systems

n/a	Involved in the study
☐	☒ Antibodies
☒	☐ Eukaryotic cell lines
☒	☐ Palaeontology and archaeology
☒	☐ Animals and other organisms
☐	☒ Clinical data
☒	☐ Dual use research of concern
☒	☐ Plants

Methods

n/a	Involved in the study
☒	☐ ChIP-seq
☒	☐ Flow cytometry
☒	☐ MRI-based neuroimaging

Antibodies

Antibodies used

Estrogen receptor [ER], clone (SP1)250; progesterone receptor [PR], clone 1E2; HER-2, clone 4B5; Ki-67, clone 30-9; pan-CK, GATA-3, clone: L50-823; HMFG, clone: SPM291; MUC1, clone: H23; CD8, clone SP57; PD-L1, clone SP263; CD20, clone L26; CD68, clone PG-M1; pan-CK, clone AE1/AE3

Validation

All antibodies excluding PD-L1 have been clinically validated and are routinely used in the clinical setting.

PD-L1 antibody landscape is The VENTANA PD-L1 (SP263) Assay is an FDA-approved CDx now used in adjacent oncological settings. There is still heavy debate on which PD-L1 antibody is best used in breast cancer and multiple antibodies have now been validated for different therapies. At the time these data were being generated,this specific antibody was chosen for logistic and price reasons.

Clinical data

Policy information about clinical studies

All manuscripts should comply with the ICMJE guidelines for publication of clinical research and a completed CONSORT checklist must be included with all submissions.

Clinical trial registration

N/A

Study protocol

The study protocol can be accessed by emailing the corresponding author.

Data collection

Patients were accrued within a 13 month period (02/2019-02/2020). Patients' medical charts were reviewed to obtain disease information, including dates of diagnoses, treatment histories, pathology reports, therapeutic histories, and clinical tumor markers. Data variables from clinical biopsies were combined with postmortem evaluation to create a visual depiction of changes over time and through disease progression in each patient.

Outcomes

Aim: Assess clinical heterogeneity of tumors within and between individuals at end-of-life and overtime.
Methods: Immunohistochemistry, scored by a board-certified clinical pathologist. Chart review for historical specimen values.
Endpoints: Expression levels for clinically relevant variables ER, PR, HER2, Ki67, PD-L1

Aim: Assess the presence of pre-malignant lesions or cancer cells within disease-free organs
Method: Immunohistochemistry, scored by a board-certified clinical pathologist.
Endpoint: Identification of cancer cells or cancer stem cells

Plants

Seed stocks

Report on the source of all seed stocks or other plant material used. If applicable, state the seed stock centre and catalogue number. If plant specimens were collected from the field, describe the collection location, date and sampling procedures.

Novel plant genotypes

Describe the methods by which all novel plant genotypes were produced. This includes those generated by transgenic approaches, gene editing, chemical/radiation-based mutagenesis and hybridization. For transgenic lines, describe the transformation method, the number of independent lines analyzed and the generation upon which experiments were performed. For gene-edited lines, describe the editor used, the endogenous sequence targeted for editing, the targeting guide RNA sequence (if applicable) and how the editor was applied.

Authentication

Describe any authentication procedures for each seed stock used or novel genotype generated. Describe any experiments used to assess the effect of a mutation and, where applicable, how potential secondary effects (e.g. second site T-DNA insertions, mosiacism, off-target gene editing) were examined.
